# Testing a multicomponent lifestyle intervention for combatting childhood obesity

**DOI:** 10.1186/s12889-021-10838-1

**Published:** 2021-04-29

**Authors:** Ivo Vlaev, Michael J. Taylor, David Taylor, Paul Gately, Laura H. Gunn, Aliza Abeles, Abdelhamid Kerkadi, Jackie Lothian, Sahar Karim Jreige, Aziza Alsaadi, Mohamed G. Al-Kuwari, Suhaila Ghuloum, Hanan Al-Kuwari, Ara Darzi, Mohamed Ahmedna

**Affiliations:** 1 Warwick Business School, University of Warwick, Coventry, UK; 2 Division of Epidemiology and Public Health, University of Nottingham, Nottingham, UK; 3 Department of Surgery and Cancer, Imperial College London, London, UK; 4 Leeds Beckett University, Leeds, UK; 5 Department of Public Health Sciences & School of Data Science, University of North Carolina at Charlotte, Charlotte, USA; 6 School of Public Health, Imperial College London, London, UK; 7 Human Nutrition Department, College of Health Sciences, Qatar University, Doha, Qatar; 8 Department of Health Sciences, Qatar University, Doha, Qatar; 9 Supreme Education Council, Doha, Qatar; 10 Aspetar Aspire, Doha, Qatar; 11 Hamad Medical Corporation, Doha, Qatar; 12 North Carolina Agricultural and Technical State University, Greensboro, USA

**Keywords:** Childhood obesity, Weight management program, Intervention, Community health, Behaviour change

## Abstract

**Background:**

Childhood obesity is a major global health concern. Weight-management camps involving delivery of a program of physical activity, health education, and healthy eating are an effective treatment, although post-intervention weight-management is less well understood. Our objective was to assess the effectiveness of a weight-management camp followed by a community intervention in supporting weight-management for overweight children and children with obesity.

**Methods:**

Participants were overweight Qatari schoolchildren or schoolchildren with obesity, ages 8–14 years, (*n* = 300) recruited over a three-year period across 14 randomly selected schools in the Doha area. They attended a two-week weight management camp, then a 10-week program of weekly lifestyle education and physical activity sessions, which also included behavior change techniques. The programme was cognitive behavioural therapy (CBT)-focused with a strong element of behavioural economics blended in.

**Results:**

Participants saw a significant BMI SDS reduction as a result of the entire intervention (camp + education and activity sessions) both at the individual (*p* < 0.0001) and cluster/school (*p* = 0.0002) levels, and weight loss occurred during each intervention stage separately for the camp (*p* < 0.0001 for both the individual and cluster/school levels) and the lifestyle education and activity phase (*p* < 0.0001 and *p* = 0.0220 at the individual and cluster/school levels, respectively).

**Conclusions:**

Weekly lifestyle education and activity sessions which include behavior change techniques may be useful in promoting continued weight management in the period following intensive, immersive childhood obesity interventions.

**Trial registration:**

ClinicalTrials.gov NCT02972164, November 23, 2016.

## Background

There has been a considerable increase in global obesity prevalence among children and adolescents in both developed and developing countries in recent decades. The 2013 global statistics on prevalence of obesity and overweight indicate that in 2013, 23.8% of boys and 22.6% of girls in developed countries, and 12.9% of boys and 13.4% of girls in developing countries were classified as children with obesity [1]. Childhood obesity increases the risk of serious health problems including hypertension, dyslipidemia, and insulin resistance [2, 3], has negative effects upon psychosocial factors such as self-esteem [4], and is associated with increased adult mortality [5].

Evidence indicates that participation in an intervention that has physical activity, dietary, and behavioral components can lead to children with obesity achieving a healthier weight [6–8]. A common form of multicomponent intervention is participation in a community program, which supports attendees to change their diet, exercise, and lifestyle behaviors [9]. Meta-analysis has indicated that although such programs often result in short-term weight-loss, the extent of weight reduction achieved tends to be relatively small [8, 10], suggesting a limited long-term effectiveness in treating severe obesity [9].

Weight-management camps are an alternative, more intensive, childhood obesity treatment, which involve attendees undergoing a daily schedule of exercise activities and healthy eating [9]. They have been shown to be effective in inducing significant short-term weight-loss for children with obesity [9, 11–14], and to have lower rates of attrition and greater weight loss extent than community interventions [15]. Weight loss achieved at weight-management camps can be sustained in the months following the intervention, but considerable variability has been revealed in the extent to which this occurs, and achievements in weight loss are generally not sustained for all attendees [15–17]. Participants of this form of intervention may, therefore, benefit from continued support after returning home. The present study investigated effectiveness of an intervention consisting of a weight management camp followed by a community intervention, consisting of weekly after-school sessions involving lifestyle education and exercise activities. The intervention took place in Qatar, where the prevalence of obesity has been estimated to be as high as 25% for children aged 10–14 [18]. Obesity prevalence appears to increase with childhood age to a peak around age 12–13 [18, 19], indicating a need for effective interventions targeting children of this age.

The camp aimed to achieve significant weight reduction, and the community sessions aimed to help to sustain the weight-loss achieved at the camp through consolidation of lifestyle education and continuation of healthy dietary and physical activity behaviors in which the children had engaged at the camp. It was hypothesized that the weight-management camp would induce weight loss, the after-school clubs would aid continued weight reduction, and that the overall intervention would result in weight-loss.

## Methods

### Study design

The study involved an intervention for which participants were invited to attend (free of charge) a two-week weight-management and lifestyle education camp (the two-week ‘camp phase’), then to attend 10 weekly lifestyle education and activity sessions, described as ‘clubs’ (the 10-week ‘club phase’), with a 3 week break between phases. For pragmatic reasons, the camp phase was delivered with four schools January 25–February 5 in 2015, five schools in January 24–February 4, 2016, and five schools in January 22–February 2, 2017; all three camp dates correspond to the Spring break for public schools in Qatar. This study followed an initial ‘pilot phase’ in 2014, in which experimental data were collected.

The primary outcome variable was body mass index standard deviation score (BMI SDS; also known as z-score). BMI was calculated using height and weight measurements recorded by members of the research team using a wall-mounted stadiometer and a calibrated Seca scale (SECA, Ohio, USA) at the camp or participants’ schools. BMI values by age and gender were used to classify obesity status of participants. Measurements were compared to World Health Organization population reference data [20] to determine BMI standard deviation scores. Measurements of participants were recorded at the beginning and end of the camp phase, as well as at the end of the club phase.

### Participants

Selection of participants involved three phases: (1) random selection of participating schools by gender (boys’ or girls’ schools) since schools in Qatar are segregated by gender, (2) invitation of all students who meet the eligibility criteria within each gender-segregated school to participate in the intervention, and (3) random selection of the required number of children participants from the list of children whose parents consented to participate.

Participant eligibility criteria were to be between the ages of 8 and 14 years at the time of recruitment and to qualify as overweight children or children with obesity, with a body mass index (BMI) on at least the 92nd percentile for their age and gender, compared to International Obesity Taskforce [21] data. Eligible children were identified using height and weight data collected by the resident school nurses, based on the same national standard protocol provided by the Ministry of Education to all schools in Qatar, at the beginning of the school year in which the intervention was to take place.

Children were recruited to the study from the school that they attended. Each school was a site of recruitment. Schools to be included in the study were randomly selected from a list of 69 in Doha and the surrounding area; this was done by an individual outside the research team who performed a random number generation for selection. Throughout the study period, 14 schools were selected for recruitment (children in Qatar attend same-sex schools and six were boys’ schools and eight were girls’ schools).

Letters inviting participants to apply to take part in the study were sent to all eligible children in every selected school. Of those wishing to take part, 25–35 from each school were randomly selected for participation with the aim of, each year, recruiting approximately 100 participants. Microsoft Excel was used to generate random numbers for this selection process.

Information about the study, consent forms, and invitations to study engagement events were delivered to parents or guardians of eligible children 3 months before the start of the camp. Parents had the opportunity to attend engagement events where information was given about the content of the camp and after school clubs, and they had an opportunity to ask any questions. These events were held before the start of the camp, during the camp, and during the club phases.

### Statistical methods

Descriptive analyses were conducted, and results were summarized by sex. In addition, six hypothesis, one-sided t-tests were performed to assess levels of performance of the intervention: (1) baseline vs. post-camp; (2) baseline vs. post-club; (3) post-camp vs. post-club – each at the individual-level (primary analysis) and cluster/school-level (sensitivity analyses). The latter sensitivity analyses were performed through: (1) one-sided t-tests on the (equally weighted) means of each school, to ensure that the results are not overly affected by outlying schools with larger numbers of participants; and (2) random effects models where overall means and school-specific random effects were included to estimate the inter-school variability across phases. These latter models are expressed as:

$$ {\displaystyle \begin{array}{l}\Delta \mathrm{BMI}\_\mathrm{SDS}\left[\mathrm{i},\mathrm{j}\right]=\mathrm{alpha}+\mathrm{mu}\ \left[\mathrm{j}\right]+\mathrm{epsilon}\ \left[\mathrm{i},\mathrm{j}\right]\\ {}\mathrm{mu}\ \left[\mathrm{j}\right]\sim \mathrm{N}\ \left(0,\mathrm{sigma}\hat{\mkern6mu} 2\right)\end{array}} $$

where ΔBMI_SDS[i,j] represents the change in BMI SDS for child i in school j, alpha is the between-phase mean intervention effect, mu [j] is the school j-specific random effect, epsilon is the child-specific error term, and the random intercepts, mu [j] follow a normal distribution with mean zero and standard deviation sigma.

Each t-test hypothesis within the primary and sensitivity analyses assessed whether that phase of the intervention was associated with an improvement in BMI SDS. A Holm adjustment was performed to account for multiple tests. Statistical analysis was conducted using R statistical software, including the lme4 package.

### Ethics, consent and permissions

Ethical approval was granted by the Qatar University Institutional Review Board; informed consent was obtained from all participants and their parents or guardians.

### Intervention descriptions

#### The camp phase

The two-week camps took place during the local school holidays, from January 25 to February 5 in 2015, January 24 to February 4 in 2016, and January 22 to February 2 in 2017. This phase aimed to provide attendees with positive healthy lifestyle experiences and to help them understand how food and activity influence weight and health.

Due to cultural norms, participants did not stay overnight at the camp; transportation by bus between participants’ homes and the camp at the start and end of the days was provided. No activities took place on Fridays, allowing children to spend time at home with their families, pray, and take part in social activities.

**Fig. 1:**
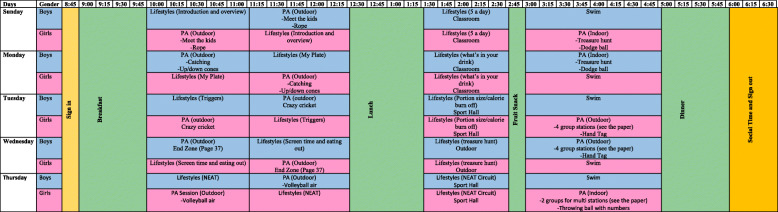
Schedule of weekly activities at the camp

On the other days, attendees took part in a structured program of lifestyle education and physical activity sessions between 09:00 and 18:00. Figure 1 shows the schedule of weekly activities at the camp. Sessions were delivered by staff with expertise in physical education and nutrition.

Physical activities were relevant to those that children would be exposed to in school and community settings including sports (football, Basketball), Exercise (aerobic activity, circuit training), Leisure (swimming as well as fun based games such as treasure hunts). Session objectives were to develop skills / competence and therefore intrinsic motivation, to engage in activities that fostered social interactions and developed relationships and also are enjoyable. These elements were in place to establish long term engagement in these activities.

Lifestyle education sessions involved teaching on health-related topics including portion control, food labeling, and awareness of sugar and fat in food and drink. Children engaged in moderate dietary restriction at the camp. Breakfast, lunch, dinner, and a snack were provided to all attendees each day in one of four portion sizes according to their height, weight, and gender. Meal planning was arranged and supervised by a nutritionist who ensured that the meals contained a healthy mixture of nutrients and food groups where meal items were color coded at a buffet line monitored by trained staff nutritionists to allow participants to mix and match the right plate items to meet the appropriate nutritional value and calorie count for their body weight class. Snacks were either fresh fruits and vegetables or snacks chosen to be either low-reduced fat and sugar such as yogurt. Meals were developed and prepared using Qatar Dietary Guidelines. Based on the guidelines, the main meals (lunch and dinner) were prepared by a resident nutritionist Chef at professional restaurant (Aspire.qa) to ensure nutritional balance.

There was no control over what children ate when they were at home in the evenings, but parents were advised that their children had already had all their meals for the day and they did not need to eat any more full meals. Parents were also advised, however that it would be appropriate for their children to eat light, healthy snacks, such as fruit, if they were hungry. Children’s food intake at home was not recorded. Parents of participants were invited to attend a graduation ceremony held after the camp to celebrate the participants’ achievements.

The programme employed Cognitive Behavioural Therapy (CBT) with a strong element of behavioural economics blended in. The CBT approach [9] includes techniques such as Education and Awareness, Stimulus Control, Monitoring / Journaling, Behaviour Shaping, Goal Setting and Planning, Problem Solving, Time Management, Social Support, and Motivational Interviewing. The Behavioural Economics approach combines lessons from psychology with those from economics by focusing on the more automatic processes of decision-making; and changing behaviour is achieved by changing the context/environment within which our decisions are taken [21]. The ‘dual process’ model has often been proposed as a unified theoretical basis for targeting rational/reflective behaviours with CBT-based approaches and automatic behaviours with contextual ‘nudges’ [22]. So, the children additionally engaged in several health-related behavior change techniques targeting automatic processes (summarized in the MINDSPACE framework for behaviour change [21]: Messenger, Incentives, Norms, Defaults, Salience, Priming, Affect, Commitments, Ego). Table 1 describes the behavior change techniques used in our intervention.

**Table 1:** Behavior change techniques used in our intervention

Technique	Description
Education and Awareness	Educational information is included to raise the participants’ awareness of specific factors that are influencing their weight and to provide examples of solutions for behaviour change.
Stimulus Control	Stimuli or cues are triggers to eating/sedentary behaviours e.g. feeling down, easy access to food, celebration events, hunger, and peer pressure. We help participants to appreciate that it is therefore important to reduce unhealthy food and sedentary cues and create an environment more conducive to long term weight management. Examples of this technique include shopping online to prevent being tempted by offers or energy dense foods; only having fruit snacks in the house; and having trainers easily accessible/visible to keep up the walking to work or school routine. Advising Qatari schools to offer healthy foods and provide shopping healthy foods tips for students. QU Human Nutrition Program students can help with this effort.
Monitoring / Journaling (starts session 1)	Monitoring of behaviours is one of the strongest behaviour change tools, as it provides feedback on progress to date. We encourage participants to monitor their behaviours regularly. The greater the detail provided, the greater the information available to make small and sustainable changes. Where feasible we shall use mobile technology tools to facilitate self-monitoring; for example, a physical activity device worn on the belt or wrist, that communicates via the family computer.
Behaviour Shaping	This involves coaching participants to help them understand how they can influence their behaviour, which is important. Examples include taking the stairs instead of escalators and lifts and/or encouraging eating a new vegetable or food at mealtimes or a different piece of fruit at snack time. Also, providing such alternative responses with positive outcomes is critical to helping behaviour change, because most behaviours tend to be a formed by previous experiences.
Goal Setting and Planning (starts session 1)	Goal-setting is an initial priority. Without a goal, an individual will be unclear about their weight loss journey. End and journey goals provide the direction of the weight management programme. Planning ensures the steps to these goals are clear and owned by the individual. In fact, all sessions involve the review and development of goals. Goals should be SMART (Specific, Measurable, Achievable, Realistic, and Time-specific): *Specific:* Specific and detailed: for example, rather than writing ‘I am not going to miss meals’ participants are encouraged to write ‘I will not miss breakfast’. *Measurable:* Participants are shown how to measure their progress and make appropriate changes. Instead of writing ‘I will walk 3 miles in 4 months.’ they are encouraged to state ‘I will walk 2 miles in 1 h in the first month increasing to 3 miles in an hour at the end of 4 months’. *Achievable:* This ensures that their goals are in reach and they have a good chance of achieving them. Instead of writing “I want to be a size 10” they would be encouraged to write “I would like to lose 3–4 pounds a week during the programme”. This is much safer and much more achievable. If goals are not achievable then they run the risk of setting themselves up to fail. This is also forgiving as it specifies “3–4 pounds” not “4 pounds” therefore if they lose 3 pounds they have still met their goal. *Realistic:* Instead of “I will give up chocolate for the rest of my life” they will be encouraged to state “This week I will cut down from a snickers bar every day to 2 snickers bars during the week.” It isn’t realistic to give up chocolate for the rest of a person’s life, and neither is it necessary. *Time specific:* Here they will be shown how to arrive at realistic time frames for goals. In weight management we should think about months and years rather than days and weeks. Instead of writing “I want to lose a stone” they will be encouraged to write “I want to lose 1–2 pounds a week for the next 12 weeks”.
Problem Solving	Weight management is difficult because life has a nasty habit of getting in the way. Each participant will be shown how to have a plan B, or a set of ready-made solutions to common barriers, as an effective way of keeping them on track. During each session, participants will be asked to consider what challenges they might face and how they can plan to overcome them. What if they forget their healthy packed lunch? What if their bike isn’t working? What if they have a bad day at work? Do they have a plan B in place for each?
Time Management	We observe in many of the individuals we work with that they have difficulty in managing their time; the outcome of this is they make rash decisions especially around eating and activity. To help, we encourage them to think about priority management rather than time management. This helps the participant think of their behaviours rather than the specific units of time they may have. This strategy is linked with monitoring, goal setting and planning so that the participant can continually manage their weight.
Social Support	This is the support provided by peers and other family member during and following attendance of the service. The majority of participants will access group support as part of their weight loss journey and during these sessions peer support should be strongly encouraged. The online community provides a further mechanism to encourage access to social support. We encourage the group to pass on details with each other so that they can have ‘support buddies’ available; and also make it clear within the sessions that building up social networks is a key objective of the programme.
Motivational Interviewing	Prompting the person to provide self-motivating statements and evaluations of their own behaviour to minimize resistance to change. Project trainers and parents play a key role in this.
Incentives	Behavior change is more likely to occur when it is immediately rewarded. Therefore, the children earned points during their time at the camp for effort, good behavior, and achievements, especially those relating to conduction of healthy behaviors.
Norms	We are strongly influenced by what others do, therefore the children were made aware of peers who had earned large numbers of points through use of leaderboards.
Salience	Our attention is drawn to what is novel and seems relevant to us. During one of the lifestyle education lessons, the children used a ‘mental contrasting’ technique, which involves being prompted to imagine a desirable outcome, to mentally contrast this outcome with their present situation, and to focus on things that they may need to change to achieve the outcome. There is evidence that using this technique can increase likelihood of positive behavior change being achieved. Specifically, the attendees of the camp wrote: (1) An aim for them to achieve relating to health (e.g. to eat more fruit and vegetables); (2), the most positive outcome of achieving their aim, and events and experiences they associate with this positive outcome (e.g. to feel happier); and (3) the most critical obstacle to their achieving this aim, together with events and experiences associated with this obstacle (e.g. no fruit at home).
Commitments	Behavior change research evidence indicates we are likely to behave in ways consistent to our public promises, therefore the children signed contracts to declare their intentions to behave in healthy ways. At the start of the camp, contracts between participants, their parents or guardians and the project team were created and signed. These outlined the goals of the three parties; these goals related to supporting the child to conduct healthy behaviors. At the start of individual daily activities at the camp, ‘group contract’ agreements were signed by all of the camp attendees involved, on a single piece of paper, to encourage them to adhere to behavioral recommendations when taking part in certain lifestyle or activity sessions.

#### The after-school club phase

A lifestyle education and weight management program was held for participants who began approximately 3 weeks after the end of the camp. Children attended school as usual during the club phase. The after-school program consisted of 10 weekly ‘club’ sessions. During the pilot year (2014), each session lasted for 2 h and took place at the children’s school, directly after their school day (see Table 2 for an example schedule). Following feedback from participants’ parents that weekend clubs would be preferable, club sessions took place on Saturdays during the full three-year study (2015–17), and lasted for four rather than 2 h. The after-school program consisted of 10 weekly ‘club’ sessions. During the pilot year (2014), each session lasted for 2 h and took place at the children’s school, directly after their school day (see Table 2 for an example schedule). Following feedback from participants’ parents that weekend clubs would be preferable, club sessions took place on Saturdays during the full three-year study (2015–17), and lasted for four rather than 2 h.

**Table 2:** Example of a schedule of an after-school club session

Time	Activity
13.30–14.00	• Meet and greet the children • After-school snack • Commencement of recording children’s anthropometric measurements
14.00–14.45	• Lifestyle lesson (the topic for this lesson changes weekly)
14.45–15.00	• Summary of key messages • Closing remarks and end of the session • Rewarding and recognizing achievements of participants, including the naming of the ‘star of the week’ • Goal setting task
15.00–16.00	• Physical activity session

Club sessions in all years aimed to support participants to consolidate their learning from the camp to increase the likelihood of their continuing the new healthy behaviors they had learned at the camp in their home environment. The curriculum was adapted from the MoreLife (UK) [22] Limited Community Club model that has been implemented to support weight management for children in the United Kingdom, and was modified to be more relevant to people living in Qatar. Adaptations include substitutions of food items with equivalent based on local food availability and habits and choice of physical activity in compliance with cultural norms such as gender segregation with same sex staffing, modest dress code, and use of activities consistent climate-related constraints. Some key modifications included:

*Gender* – There were clear requirements to separate children based on their sex, this required modifications to the length and timings of the programme as well as staffing requirements.

*Prayer* – Time and appropriate locations was set aside so that children could pray. Fridays were excluded from any program activities as the day represents a religious weekend day.

*Content* – The original MoreLife curriculum was reviewed by the programme team and modifications we made to ensure cultural appropriateness. For example, foods that are commonly consumed in Qatar were used as examples, culturally appropriate events Ramadan were discussed.

*Meals* – The typical meal programme was based at the MoreLife camp but was modified with a team of local representatives (including the Chef at our programme location, and registered dietician members of the team), traditional and common local dietary practices for children in Qatar were provided. Food items for Morelife options that are culturally or religiously unacceptable were substituted with their local equivalent.

*Activity* – The physical activity programme was broad and included activities that would be available to local Qatari children. Consideration was also given to gender, dress code, and the local climate. For instance, girls had to be trained with only female staff members and all had to dress modesty consistent with the local norms.

The sessions were delivered by staff with expertise in physical education and nutrition, and consisted of lifestyle and diet education, physical education, and social and team-building activities (see Table 3). Height and weight measurements were recorded throughout the duration of the program to ensure that weight-loss was taking place safely.

**Table 3:** Curriculum of the after-school clubs

Session number	Topic of lifestyle education	Topic of physical education
1	Introduction and setting the scene	Team Building
2	Portion control	Multi skills
3	Eat-well Plate	Circuits
4	Food Preparation – Eat-well	Football
5	Food Labels	Basketball
6	Fat content in Food	Choice physical activity
7	Eating out, fast-food, and takeaways	Rounders
8	Importance of physical activity and reducing sedentary behaviors	Dance
9	Recipe planning: Healthy options	Dodgeball, Boxercise
10	What have I learnt, and next steps	Choice physical activity

During the after-school club, we used the same behavior change techniques employed at the weight management camp (the CBT plus behavioral economics approaches described above).

To ensure continued children buy in and long term success, parental engagement continued throughout the intervention phases and included (1) parent orientation before camp, (2) “Coffee with parents” sessions during camp where a dietician delivered information ways to continue the healthy plan at home after camp day, (3) a series of parental workshops during the club phase to enhance parental knowledge in nutrition and healthy lifestyle, (4) invitation to parents to visit the clubs at their own time to learn about the after school club program and ways to support their child, (5) WhatsApp parental group list through which parents received tips from intervention manager on helping their children practice and sustain healthy habits practiced at the after school clubs.

## Results

**Fig. 2:**
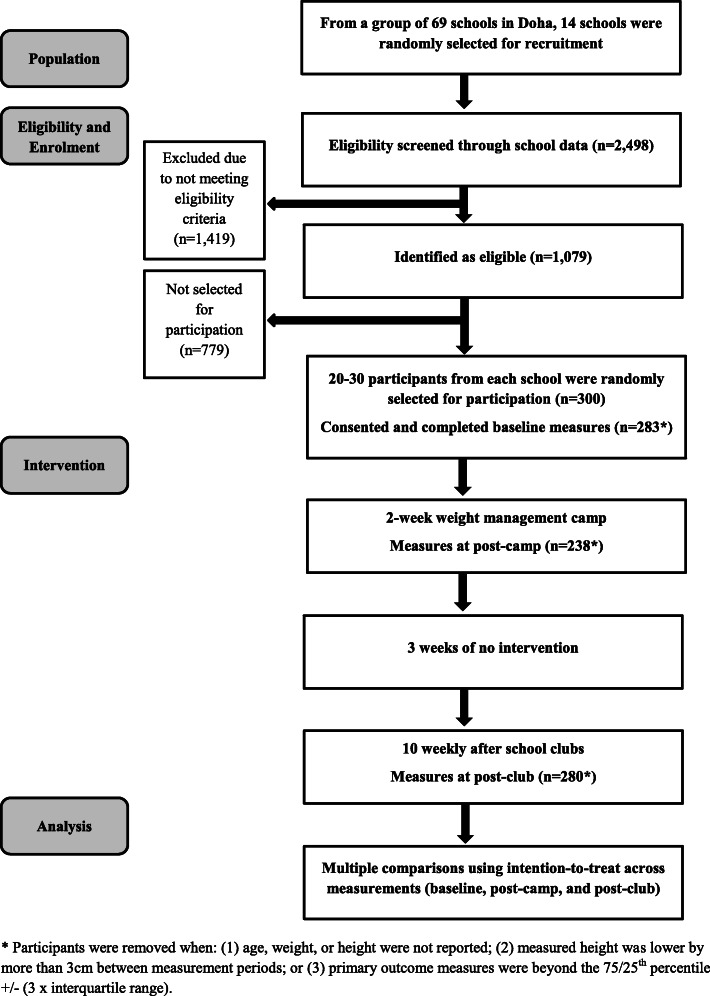
Flow of participation in the trial

**Fig. 3:**
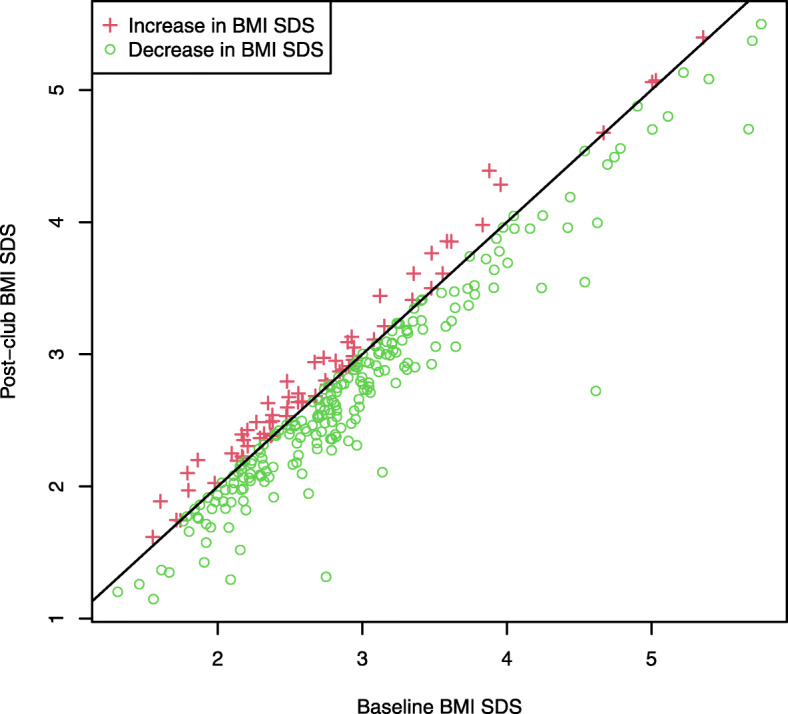
Change in BMI SDS between baseline and post-club, with the diagonal line representing no change

Figure 2 shows flow of participation. There were 300 participants across 14 schools in the study. Those who were missing height or weight values at baseline (*n* = 14) were removed from analysis. Date of birth was complete across all participants. Participants whose BMI SDS measurements indicated changes beyond the 75th percentile + (3 multiplied by the interquartile range) or below the 25th percentile – (3 multiplied by the interquartile range) throughout the study were identified as having potentially erroneous outlying measurements and were removed from analysis. This resulted in the removal of one participant from the study at baseline. Hence, at baseline, there were valid measurements for 285 participants (143 females) aged 8–14 years (M 10.61 SD 1.05) with a mean BMI SDS of 2.90 (SD 0.83). Table 4 shows descriptive statistics for baseline, post-camp, and post-club measurements. Figure 3 further illustrates the BMI SDS changes from baseline to post-club.

**Table 4:** Descriptive Statistics at Baseline, Post-camp, and Post-club Measurements

Descriptive Statistics	Total (*N* = 300)			Boys (*N* = 148)			Girls (*N* = 152)		
	Base-line	Post-Camp	Post-Club	Base-line	Post-Camp	Post-Club	Base-line	Post-Camp	Post-Club
N with age, height, and weight recorded	286	240	284	143	115	140	143	125	144
N for BMI SDS recorded after removal of 1 outlying/incorrect record	285	239	283	142	114	139	143	125	144
Mean (SD) BMI SDS assuming ITT^a^	2.90 (0.83)	2.82 (0.81)	2.76 (0.81)	3.09 (0.88)	3.02 (0.84)	2.97 (0.85)	2.72 (0.73)	2.62 (0.73)	2.56 (0.71)
Mean (SD) BMI SDS, per child measured, at each of the three measurement stages		2.77 (0.79)	2.76 (0.81)		2.98 (0.82)	2.96 (0.85)		2.58 (0.71)	2.56 (0.72)

^a^For children with valid observations at baseline, and assuming intention-to-treat (zero changes in BMI SDS if not recorded) from baseline when follow-up measurement observations are not available

### Comparison of overall effect of intervention from baseline to post-club

To analyze the effect of the intervention both at the individual and cluster/school levels, mean BMI SDS change of participants was calculated using an intention to treat design with ten participants lost to follow-up assumed to have had no change in BMI SDS. Note that two additional participants were removed (see Table 4), since their height values from baseline to post-club measurements showed a shrinkage greater than 3 cm, which would also be indicative of erroneous measurements (rather than reasonable measurement errors or changes in shoe thickness), yielding a sample size at baseline of *n* = 283 upon which analyses were performed. Table 4 displays results of paired t-tests comparing the means, which revealed a significant effect of the intervention both at the individual level (*t* (282) = − 9.34, *p* < 0.0001 [Mean difference = − 0.14; 95% CI -0.17, − 0.11]) and school level (*t* (13) = − 4.78, *p* = 0.0002 [Mean difference = − 0.15; 95% CI -0.21, − 0.08]) upon the extent of BMI SDS change.

### BMI SDS change during the camp

BMI SDS measurements were recorded from 283 participants at the start of the camp at baseline with an intention to treat assumption. Table 5 shows results of analyses indicating a significant BMI SDS reduction both at the individual level (Mean difference = − 0.09, 95% CI -0.11, − 0.08; *t* (282) = − 11.30, *p* < 0.0001, from a mean of 2.91 (SD 0.82) to 2.82 (SD 0.81)) and cluster/school level (Mean difference = − 0.09, 95% CI − 0.13 to − 0.05; *t* (13) = − 5.24, *p* < 0.0001).

**Table 5:** Paired T-tests for BMI SDS, at the child and cluster/school levels, for six measurement comparisons using a Holm adjustment for multiple tests

Paired T-tests (Child level – Primary analysis)	Mean/Estimate^**a**^	SD/SE^**a**^	95% CI	t-stat	***p***-value
Baseline to Post-Camp	-0.09	0.13	(−0.11,-0.08)	−11.30	< 0.0001
Baseline to Post-Club	− 0.14	0.26	(− 0.17,-0.11)	−9.34	< 0.0001
Post-Camp to Post-Club	−0.06	0.25	(−0.09,-0.03)	−3.79	< 0.0001
**Paired T-tests (School level – Sensitivity analysis)**					
Baseline to Post-Camp	−0.09	0.07	(−0.13,-0.05)	−5.24	< 0.0001
Baseline to Post-Club	−0.15	0.12	(−0.21,-0.08)	−4.78	0.0002
Post-Camp to Post-Club	−0.07	0.11	(−0.13,-0.002)	−2.23	0.0220
**Random Effects Models (Sensitivity analysis)**					
Baseline to Post-Camp					
alpha	−0.09	0.02	(−0.13,-0.06)	−5.15	< 0.0001
sigma	0.06		(0.04,0.09)		
Baseline to Post-Club					
alpha	−0.15	0.03	(− 0.21,-0.08)	−4.82	< 0.0001
sigma	0.10		(0.05,0.16)		
Post-Camp to Post-Club					
alpha	−0.06	0.03	(−0.12,-0.01)	−2.32	0.0102
sigma	0.08		(0.03,0.14)		

^a^Means and standard deviations (SDs) correspond to t-tests, while model estimates and standard errors (SEs) correspond to random effects models

### BMI SDS change during the after-school clubs

**Fig. 4:**
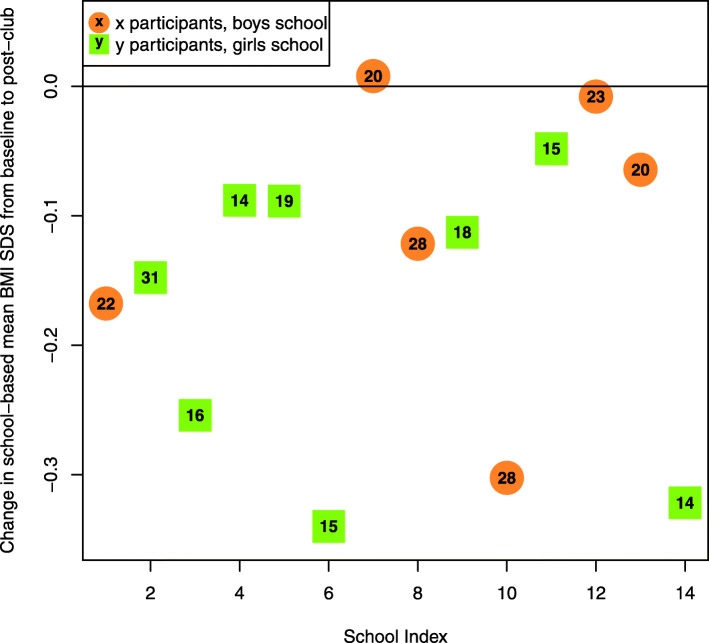
Mean changes in BMI SDS by school between baseline and post-club, including sex and within-school sample size. Negative numbers indicate reductions from baseline to post-club follow-up

BMI SDS measurements were recorded for 238 participants from the end of the camp to the end of the club, assuming an intention to treat analysis (see Table 4) [note that this is based on one participant who was removed since their height values from post-camp to post-club measurements showed a shrinkage greater than 3 cm]. Table 5 shows that a statistically significant BMI SDS reduction was found to occur during the after-school club stage of the intervention both at the individual level (Mean difference = − 0.06, 95% CI − 0.09, − 0.03; *t* (237) = − 3.79, *p* < 0.0001, from a mean of 2.77 (SD 0.79) to 2.71 (SD 0.79)) and cluster/school level (Mean difference = − 0.07, 95% CI -0.13, − 0.002; *t* (13) = − 2.23, *p* = 0.0220). Table 5 also includes results of the random effects models of the sensitivity analysis in which the inter-school variability parameter, sigma, is estimated for each BMI SDS change between intervention phases. For example, from baseline to post-club, the estimated mean reduction in BMI SDS is 0.15 (95% CI -0.21, − 0.08, *p* < 0.0001), with an inter-school standard deviation estimate of 0.10 (95% CI 0.05, 0.16). Figure 4 further portrays the mean changes in BMI SDS by school between baseline and post-club, including sex and within-school sample size.

## Discussion

The finding that significant BMI SDS reduction occurred for the intervention overall, and occurred during both the camp and club phases suggests these stages of the intervention were effective in inducing weight loss, and that community interventions such as weekly lifestyle education sessions can be effective in supporting weight-management following an intensive childhood obesity intervention. The mean BMI SDS reductions over the camp phase and club phase of the intervention achieved by participants was 0.09 and 0.06, respectively; this indicates that many intervention participants achieved clinically relevant weight-loss as a result of the camp and/or club, as recent evidence indicates that even BMI SDS reductions smaller than 0.10 are associated with clinically significant reductions in insulin and total cholesterol [23]. The BMI SDS reduction over the entire intervention period (including camp and club) was 0.14 when assuming intention to treat between baseline and post-club measurements.

Weight-loss was achieved during the club phase despite the children having only four contact hours per week with the staff delivering the intervention, indicating that their lifestyle behaviors in the home and school environment were amenable to successful weight-management.

Although the present study gave parents of participants the opportunity to attend engagement events, it may be beneficial for future similar interventions to involve parents more centrally, with data being collected on efficacy of parent education sessions. Evidence suggests that involving families, as well as the children themselves, in a childhood obesity intervention is likely to increase the chance of intervention success [10, 24–29].

Future interventions could also assess participants’ self-esteem. Childhood obesity has been associated with lower self-esteem [4, 30, 31] and weight loss interventions for children with obesity have been associated with improvements in self-esteem [32, 33]. A limitation of this study is that no consistent measurements were observed between the end of the camp and beginning of the club phases in order to assess what occurs during an approximately 3-week gap period; and, club measurements are based on post-camp to post-club measures, which includes this short gap between intervention phases. It is unclear whether there is any weight gain occurring during this gap. If so, then there could be the potential challenge of sustaining weight-loss achieved during intense interventions followed by community interventions; and, it may be that future similar interventions could reduce any such weight gain, if it exists, through more intensive education and engagement of attendees’ families. Though, the former was not the case in research on the long-term effects of a weight management camp in the UK [16]. However, there are some important differences between this previous intervention and the present study, including that participants in the UK intervention attended the camp for up to 6 weeks, and it was residential, providing further opportunities for social engagement, whereas the camp in the present study lasted 2 weeks, and children went home to their families in the evenings and on Fridays. The relatively short duration of the camp in the present study, and therefore lack of time to form new habits, may also help to explain why extent of BMI SDS reduction was relatively similar in the camp and the club phases, whereas community interventions typically result in less weight loss than more intensive interventions [9, 10]. The extent of weight loss success during the club phase may have also been enhanced due to the participants having recently successfully altered their diet and exercise behaviors to achieve weight loss during the camp phase.

A further limitation of this study is the relatively low levels of attendance observed in the after-school club phase of the intervention; the median number of sessions attended was 5.0 of a possible 10. Another limitation is that no covariates/confounding factors were considered. While additional covariates could be useful to explore individual-level changes, this does not affect the validity of the population-level results of the study. We expect that, despite these limitations, the findings that the camp and club stages separately, and the intervention overall, were successful in inducing weight-loss for overweight children and children with obesity are generalizable to other settings.

## Conclusions

The entire intervention, as well as the weight-management camp and the after-school club phases when considered separately, were associated with significant BMI SDS reductions. Community interventions, such as the weekly after-school clubs used in the present study, may be an effective way of providing support to overweight children and children with obesity for continued weight loss after intensive weight-management camps. Future research could investigate relative effectiveness, and cost-effectiveness, of interventions involving a weight-management camp followed by a less intensive community phase in achieving long-term weight reduction for overweight children and children with obesity, in comparison to a weight-management camp, or community intervention alone.

## Data Availability

The datasets analysed during the current study are available from the corresponding author on reasonable request.

## Abbreviations

CI: Confidence interval
M: Mean
SD: Standard deviation
BMI SDS: Body mass index standard deviation score
t: t test statistic
*η*_*p*_^*2*^: Partial eta squared

## References

[1] Ng M, Fleming T, Robinson M, Thomson B, Graetz N, Margono C (2014). Global, regional and national prevalence of overweight and obesity in children and adults 1980–2013: A systematic analysis. Lancet (London, England).

[2] Daniels S (2009). Complications of obesity in children and adolescents. Int J Obes.

[3] Pulgarón ER (2013). Childhood obesity: a review of increased risk for physical and psychological comorbidities. Clin Ther.

[4] Rankin J, Matthews L, Cobley S, Han A, Sanders R, Wiltshire HD (2016). Psychological consequences of childhood obesity: psychiatric comorbidity and prevention. Adolesc Health Med Ther.

[5] Kostas K, Alexandra S, Kyriaki K (2018). Childhood obesity and its associations with morbidity and mortality in adult life. Diabetes Compl.

[6] Elvsaas I, Giske L, Fure B, Juvet LK (2017). Multicomponent lifestyle interventions for treating overweight and obesity in children and adolescents: a systematic review and meta-analyses. J Obes.

[7] Al-Khudairy L, Loveman E, Colquitt JL, Mead E, Johnson RE, Fraser H, et al. Diet, physical activity and behavioural interventions for the treatment of overweight or obese adolescents aged 12 to 17 years. Cochrane Database Syst Rev. 2017;6:CD012691. 10.1002/14651858.CD012691 PMC6481371 28639320

[8] Mead E, Brown T, Rees K, Azevedo LB, Whittaker V, Jones D, et al. Diet, physical activity and behavioural interventions for the treatment of overweight or obese children from the age of 6 to 11 years. Cochrane Database Syst Rev. 2017;6:CD012651. 10.1002/14651858.CD012651 PMC6481885 28639319

[9] Gately PJ, Haslam DW, Sharma AM, Le Roux CW (2014). Residential weight loss camps for children and young people. Controversies in obesity.

[10] Oude Luttikhuis H, Baur L, Jansen H, Shrewsbury VA, O'Malley C, Stolk RP, et al. Interventions for treating obesity in children. Cochrane Database Syst Rev. 2009;(1):CD001872. 10.1002/14651858.CD001872.pub2 19160202

[11] Wong WW, Barlow SE, Mikhail C, Wilson TA, Hernandez PM, Shypailo RJ, Abrams SH (2013). A residential summer camp can reduce body fat and improve health-related quality of life in obese children. J Pediatr Gastroenterol Nutr.

[12] Taylor MJ, Arriscado D, Vlaev I, Taylor D, Gately P, Darzi A (2016). Measuring perceived exercise capability and investigating its relationship with childhood obesity: a feasibility study. Int J Obes.

[13] Gately PJ, Cooke CB, Barth JH, Bewick BM, Radley D, Hill AJ (2005). Children's residential weight-loss programs can work: a prospective cohort study of short-term outcomes for overweight and obese children. Pediatrics..

[14] Huelsing J, Kanafani N, Mao J, White NH (2010). Camp jump start: effects of a residential summer weight-loss camp for older children and adolescents. Pediatrics..

[15] Kelly K, Kirschenbaum D (2011). Immersion treatment of childhood and adolescent obesity: the first review of a promising intervention. Obes Rev.

[16] Gately PJ, Cooke CB, Butterly RJ, Mackreth P, Carroll S (2000). The effects of a children's summer camp programme on weight loss, with a 10 month follow-up. Int J Obes.

[17] Fonseca H, Palmeira AL, Martins S, Ferreira PD (2014). Short-and medium-term impact of a residential weight-loss camp for overweight adolescents. Int J Adolesc Med Health.

[18] Al-Thani M, Al-Thani A, Alyafei S, Al-Chetachi W, Khalifa S, Ahmed A (2018). The prevalence and characteristics of overweight and obesity among students in Qatar. Public Health.

[19] Kerkadi A, Hassan AS, Eltayeb M, Yousef A (2009). High prevalence of the risk of overweight and overweight among Qatari children ages 9 through 11. Nutr Food Sci.

[20] Freeman J, Cole T, Chinn S, Jones P, White E, Preece M (1995). Cross sectional stature and weight reference curves for the UK, 1990. Arch Dis Child.

[21] Public Health England. International Obesity Taskforce 2013 [Available from: http://www.sepho.org.uk/viewResource.aspx?id=13022.

[22] MoreLife (UK) Ltd. MoreLife: Giving people more confidence, more health, more energy and ultimately, more life. 2014 [Available from: http://www.more-life.co.uk.

[23] Kolsgaard ML, Joner G, Brunborg C, Anderssen SA, Tonstad S, Andersen LF (2011). Reduction in BMI z-score and improvement in cardiometabolic risk factors in obese children and adolescents. The Oslo Adiposity Intervention Study-a hospital/public health nurse combined treatment. BMC Pediatr.

[24] Kalavainen M, Korppi M, Nuutinen O (2007). Clinical efficacy of group-based treatment for childhood obesity compared with routinely given individual counseling. Int J Obes.

[25] Hughes AR, Stewart L, Chapple J, McColl JH, Donaldson MD, Kelnar CJ (2008). Randomized, controlled trial of a best-practice individualized behavioral program for treatment of childhood overweight: Scottish childhood overweight treatment trial (SCOTT). Pediatrics..

[26] Golley RK, Magarey AM, Baur LA, Steinbeck KS, Daniels LA (2007). Twelve-month effectiveness of a parent-led, family-focused weight-management program for prepubertal children: a randomized, controlled trial. Pediatrics..

[27] McGovern L, Johnson JN, Paulo R, Hettinger A, Singhal V, Kamath C, Erwin PJ, Montori VM (2008). Treatment of pediatric obesity: A systematic review and meta-analysis of randomized trials. J Clin Endocrinol Metab.

[28] Lloyd-Richardson EE, Jelalian E, Sato AF, Hart CN, Mehlenbeck R, Wing RR (2012). Two-year follow-up of an adolescent behavioral weight control intervention. Pediatrics..

[29] Kelleher E, Davoren MP, Harrington JM, Shiely F, Perry IJ, McHugh SM (2017). Barriers and facilitators to initial and continued attendance at community-based lifestyle programmes among families of overweight and obese children: a systematic review. Obes Rev.

[30] Griffiths LJ, Parsons TJ, Hill AJ (2010). Self-esteem and quality of life in obese children and adolescents: a systematic review. Int J Pediatr Obes.

[31] Strauss RS (2000). Childhood obesity and self-esteem. Pediatrics.

[32] Lowry KW, Sallinen BJ, Janicke DM (2007). The effects of weight management programs on self-esteem in pediatric overweight populations. J Pediatr Psychol.

[33] McGregor S, McKenna J, Gately P, Hill AJ (2016). Self-esteem outcomes over a summer camp for obese youth. Pediatric Obes.

